# Galloylation-Driven Anchoring of the Asp325-Asp336 Ridge: The Molecular Logic Behind the Superior Kinetic Stabilization of HMPV Fusion Protein by Green Tea Dimeric Catechins

**DOI:** 10.3390/molecules31050821

**Published:** 2026-02-28

**Authors:** Shrikant S. Nilewar, Santosh S. Chobe, Amruta D. Gurav, Salman B. Kureshi, Srushti B. Palande, Jesica Escobar-Cabrera, Fabiola Hernández-Rosas, Tushar Janardan Pawar

**Affiliations:** 1 Department of Pharmaceutical Chemistry, Maliba Pharmacy College, Uka Tarsadia University, Bardoli 394350, Gujrat, India; shrinilewar@gmail.com; 2 Department of Chemistry, M.G.V.’s Loknete Vyankatrao Hiray, Arts, Science and Commerce College, Panchavati, Nashik 422003, Maharashtra, India; chobess222@gmail.com; 3 School of Pharmaceutical Science, Sandip University, Nashik 422213, Maharashtra, India; amrutagurav70@gmail.com (A.D.G.); salmankureshi216@gmail.com (S.B.K.); srushti.palande1704@gmail.com (S.B.P.); 4 Facultad de Química, Universidad Autónoma de Querétaro, Querétaro 76010, Mexico; jesica.escobar@uaq.mx (J.E.-C.); fabiola.hernandezro@anahuac.mx (F.H.-R.); 5 Centro de investigación, Universidad Anahuac Querétaro, Circuito Universidades I, Fracción 2 S/N, Zibatá, El Marqués, Querétaro 76246, Mexico; 6 Dirección de Mecatrónica, Universidad Politécnica de Querétaro, Carretera Estatal 420 S/N, El Rosario, Querétaro 76240, Mexico; 7 Escuela de Ingeniería Química, Universidad Anahuac Querétaro, Circuito Universidades I, Fracción 2 S/N, Zibatá, El Marqués, Querétaro 76246, Mexico

**Keywords:** molecular dynamics (MD), free energy landscape (FEL), MM/GBSA, human metapneumovirus, galloylation, fusion-locking agents, drug design

## Abstract

The human metapneumovirus (HMPV) Fusion (F) glycoprotein is a high-priority target for “fusion-locking” agents that stabilize its metastable prefusion state. While monomeric catechins like EGCG are known antivirals, the molecular basis for the superior activity of structurally complex dimeric catechins remains poorly understood. We employed an advanced biophysical workflow, integrating 100 ns all-atom molecular dynamics (MD), free energy landscape (FEL) analysis, and MM/GBSA thermodynamic integration to decode the Structure–Dynamics Relationship (SDR) of 210 *Camellia sinensis* (Green tea) phytochemicals. The results reveal a “Galloylation-Driven Anchoring” mechanism: the galloyl moiety of prodelphinidin A2 3′-gallate provides critical electrostatic complementarity to the Asp325-Asp336 acidic ridge. FEL analysis quantitatively demonstrates that this anchoring leads to pronounced stabilization of the F protein in a deep, kinetically favored global minimum (Δ*G* = 9.357 kJ/mol), effectively raising the energy barrier for the fusogenic conformational shift. This study provides a comparative and mechanistically informed computational proof-of-concept for the use of dimeric natural scaffolds as precision fusion-locking agents, offering a roadmap for experimental biophysical validation. In this workflow, molecular docking was employed exclusively as a qualitative structure-based filtering step, while all quantitative conclusions regarding stabilization and binding energetics were derived from post-docking MD, FEL, and MM/GBSA analyses.

## 1. Introduction

The human metapneumovirus (HMPV), a member of the Pneumoviridae family, has been identified as a leading cause of acute respiratory infections (ARI) globally [1]. Since its discovery in 2001 [2], HMPV has been shown to cause significant morbidity in vulnerable populations, including infants, young children, older adults, and immunocompromised individuals, often presenting with clinical symptoms indistinguishable from respiratory syncytial virus (RSV). The global burden of HMPV infection remains substantial, contributing to the annual cycle of bronchiolitis and pneumonia, which places a severe strain on public health systems. Despite this persistent and critical health challenge, therapeutic options are strikingly limited: there are currently no FDA-approved vaccines or specific antiviral medications available for the treatment of HMPV infection [3,4,5]. This deficit in the therapeutic arsenal underscores the urgent and sustained need for innovative drug discovery efforts targeting the fundamental machinery of viral entry and replication.

The most promising therapeutic intervention point for enveloped respiratory viruses lies in disrupting the viral entry mechanism, primarily mediated by the metastable Fusion (F) glycoprotein [3]. The HMPV F protein functions as the central molecular machine, undergoing a critical, irreversible conformational change from a highly energized prefusion state (the infective form) to a stable postfusion state (the inactive form) to drive the merger between the viral and host cell membranes [3,5,6]. The prefusion conformation, specifically the ectodomain structure (PDB ID: 5WB0), is the biologically relevant target, as it facilitates initial host cell attachment, notably through interactions with cell-surface glycans like heparan sulphate and sialic acid [7,8]. Stabilization of this prefusion state is a well-established mechanism for neutralization and inhibition; by locking the F protein into its metastable, pre-transition structure, the energy release necessary for membrane fusion is blocked, effectively disarming the virus before cellular entry can occur [7]. This mechanistic strategy, identifying small molecules that act as fusion-locking agents, represents a validated and highly desirable avenue for drug development against HMPV and related pathogens [6]

A compelling historical precedent for targeting viral glycoproteins with small molecules is found within the diverse chemical space of natural products [9,10,11,12]. Among these, polyphenols derived from *Camellia sinensis* (green tea) stand out, particularly (-)-Epigallocatechin-3-gallate (EGCG) [13]. EGCG is a broadly recognized antiviral agent demonstrating effectiveness against a variety of enveloped viruses, including influenza, HIV, and coronaviruses [14,15,16,17,18,19]. Its mechanism of action often involves blocking the initial stages of infection by preventing viral attachment to host glycans, a mechanism highly pertinent to HMPV F protein function [20]. Previous computational studies have recognized EGCG and its analogues as potential candidates for HMPV F protein inhibition based on docking scores [13,20]. However, the *C. sinensis* phytocomplex offers a far richer landscape of potential scaffolds beyond monomeric EGCG, including a family of structurally complex catechin dimers and galloylated derivatives known for their increased size, polarity, and potential for polyvalent interaction. Tapping into this unexplored chemical space, particularly utilizing the multi-valent binding potential of these dimeric structures, offers a systematic route to identifying inhibitors with enhanced affinity and stability [15,21,22,23].

Despite the structural characterization of the HMPV prefusion F glycoprotein (PDB ID: 5WB0) and its clear druggability, the systematic molecular modeling search for highly stable inhibitors remains incomplete [7]. Critically, previous in silico efforts, while foundational, have relied heavily on static docking simulations. While docking is essential for rapid initial triage, it represents only a single, optimized snapshot of the protein-ligand interaction, fundamentally neglecting the biological reality of protein flexibility, solvent effects, and the dynamic stability required to maintain inhibition over time [21,22,23]. While several candidates show high affinity in static models, they often fail due to a lack of kinetic persistence within the Fusion glycoprotein’s metastable state; thus, identifying leads with enduring inhibitory potential requires a transition from affinity-based screening to kinetic and thermodynamic validation. Therefore, the essential knowledge gap is not merely the absence of a potent lead, but the lack of thermodynamic and dynamic validation to confirm whether promising dock scores translate into a stable, enduring inhibitory complex under near-physiological conditions. High-quality computational chemistry demands that preliminary docking predictions be rigorously challenged by all-atom molecular dynamics (MD) simulations and subsequent binding free energy calculations (MM/GBSA or MM/PBSA) to establish computational rigor and confidence in the mechanistic findings.

**Figure 1:**
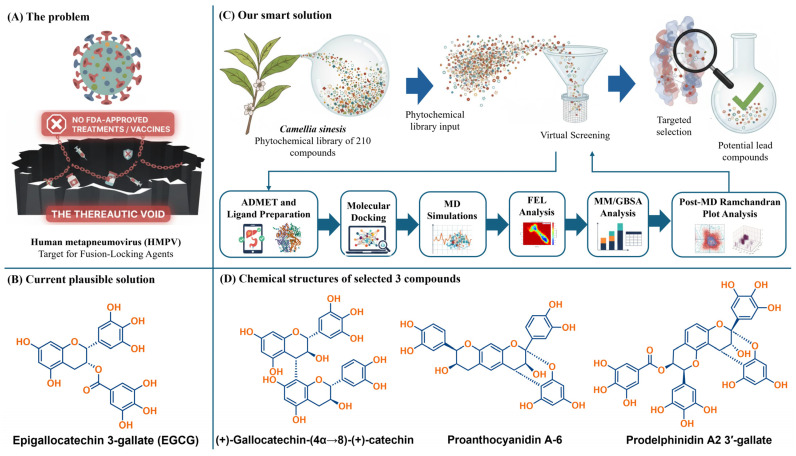
Targeted Design Strategy: Phytochemical Leads as HMPV Fusion-Locking Agents. The schematic illustrates the project rationale: moving beyond monomeric EGCG (**B**) using a multi-criteria computational workflow (**C**) to identify structurally complex dimeric catechins (**D**). These compounds are designed to prevent viral entry by anchoring and stabilizing the HMPV Fusion Glycoprotein (**A**) in its prefusion state.

This study was designed to bridge this crucial gap by deploying an integrated and rigorous computational workflow to characterize the structural and thermodynamic determinants of a comprehensive library of *C. sinensis* phytochemicals against the HMPV F prefusion ectodomain (PDB ID: 5WB0). Our strategy moves beyond the limitations of simple docking by introducing a multi-step analytical framework for lead prioritization. First, a library of 210 phytochemicals was evaluated to identify candidates with superior theoretical binding stabilization compared to EGCG and optimized ADMET properties (oral bioavailability, CNS penetration, and toxicity), selecting three highly promising catechin dimers—(+)-gallocatechin-(4α→8)-(+)-catechin, proanthocyanidin A-6, and prodelphinidin A2 3′-gallate—for in-depth biophysical modeling (Figure 1). Second, and central to the computational novelty of this work, the kinetic persistence of these three lead-protein complexes was probed over 100 ns of all-atom MD simulation followed by post-dynamic stereochemical assessment. Third, the true thermodynamic driving forces behind binding were quantified using MM/GBSA and MM/PBSA methods to determine the most thermodynamically favored lead and delineate the specific molecular logic of electrostatic, van der Waals, and solvation energy contributions.

Through this methodical approach, this manuscript provides not only a set of robust leads but also establishes a definitive molecular-level mechanistic understanding of how dimeric catechin structures interact with and stabilize the key Asp325-Asp336 ridge and loop regions, thereby elucidating the structural basis of their function as potent HMPV fusion-locking agents. The high-quality, reproducible nature of the computational modeling, incorporating MD convergence, thermodynamic correlation, Free Energy Landscape (FEL) analysis, and structural validation, provides a comparative biophysical framework for hypothesis-driven investigation. These findings are sufficient to propose these molecules as verified candidates for subsequent experimental binding and functional assays, serving as a predictive roadmap for targeting Class I viral fusion proteins.

## 2. Results and Discussion

### 2.1. Theoretical Binding Stabilization and Multi-Criteria ADMET Filtration of Phytochemicals

To initiate the identification of high-potential HMPV fusion inhibitors, a comparative biophysical assessment was conducted on a library of 210 *C. sinensis*-derived phytochemicals against the HMPV F protein prefusion conformation (PDB ID: 5WB0). The primary objective was to prioritize molecular leads using docking scores as a qualitative indicator of favorable geometric complementarity and interaction potential relative to the reference compound EGCG, which showed a binding score of −8.6 kcal/mol.

**Figure 2:**
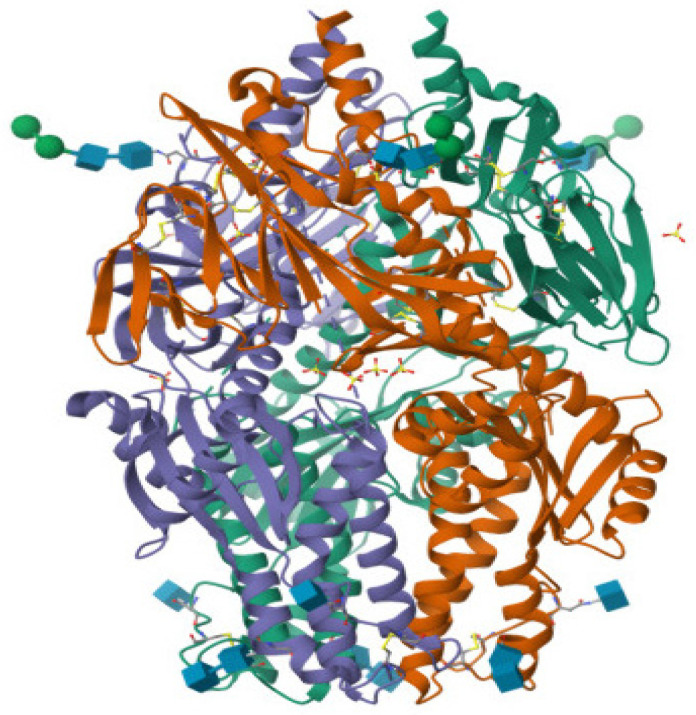
Crystal Structure of the HMPV Fusion Glycoprotein Stabilized in its Prefusion State (PDB ID: 5WB0).

**Figure 3:**
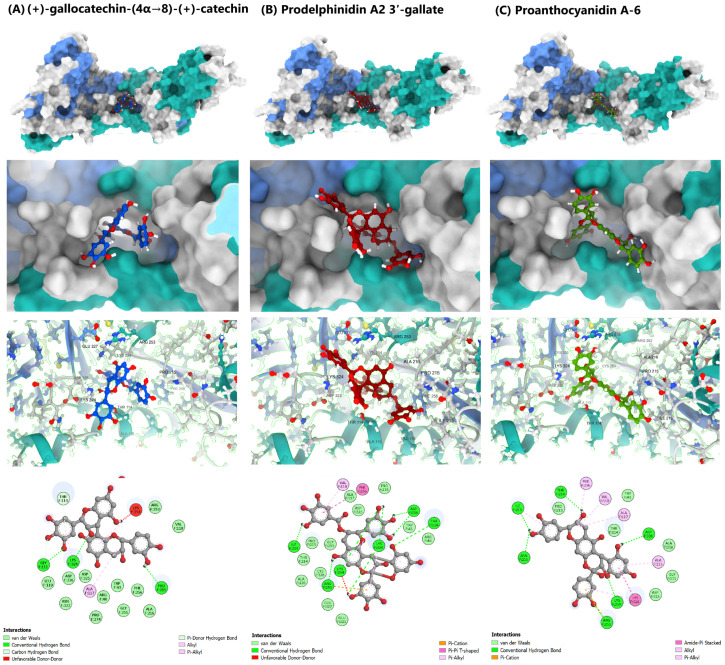
Molecular Docking Visualization of the Three Catechin Dimer Leads within the HMPV F Glycoprotein Binding Cavity. The figure illustrates the predicted binding modes and key residue contacts for (+)-gallocatechin-(4α→8)-(+)-catechin (blue), prodelphinidin A2 3′-gallate (red), and proanthocyanidin A-6 (green).

The docking results (Figure 2) confirmed that dimeric and galloylated derivatives exhibited stronger predicted affinities, with the top compounds scoring from −9.2 kcal/mol to −10.0 kcal/mol. This initial screen successfully prioritized compounds with structural features, multiple hydroxyl groups and extended aromatic systems that favor multi-point engagement within the F protein cavity. Three compounds emerged as the most promising based on favorable docking-ranked interaction potential: (+)-gallocatechin-(4α→8)-(+)-catechin (highest affinity: −10.0 kcal/mol), prodelphinidin A2 3′-gallate (−9.4 kcal/mol), and proanthocyanidin A-6 (−9.2 kcal/mol). Figure 3 provides the visualization of the predicted binding modes and interaction profiles of these selected leads (See also, Figures S4–S9, Supplementary Materials).

The interaction profile for (+)-gallocatechin-(4α→8)-(+)-catechin highlighted a dense hydrogen-bonding network (GLY111, LYS324, and PRO215) complemented by hydrophobic packing (ALA117 and THR114), leveraging its flexible B-type interflavan linkage. Prodelphinidin A2 3′-gallate, an A-type dimer containing a galloyl group, showed strong polar anchoring via hydrogen bonds to key acidic residues (LYS254 and ASP336) and aromatic stabilization (π-π T-shaped with PHE256). Conversely, proanthocyanidin A-6 relied on its rigid A-type scaffold to maintain hydrophobic and π-cation interactions, primarily stabilizing the 210–214 loop region and ASP336.

Crucially, the selection was finalized using a stringent ADMET filtration step to ensure pharmacokinetic viability, moving beyond affinity alone. This is essential for translational studies, even in the *in silico* phase. The ADMET assessment (Table 1) revealed differential safety and permeability profiles among the three top-scoring compounds:

(+)-gallocatechin-(4α→8)-(+)-catechin was selected for its comparatively balanced safety profile within this chemical class, despite recognized limitations in permeability associated with its size and polarity: low hERG cardiotoxicity and low genotoxic risk (Ames). Although its size (MW 594.14 Da) and polarity (TPSA 240.99 Å^2^) are high, it showed the most realistic Central Nervous System (CNS) exposure profile, which is beneficial for a systemic antiviral.

Prodelphinidin A2 3′-gallate was prioritized despite its larger size (MW 760.13 Da) due to its exceptionally low P-gp substrate likelihood and high predicted passive permeability (PAMPA 0.94), suggesting its transport may overcome its higher polarity. Its low cardiac risk (hERG) was a favorable factor, though a higher Ames score suggested an area for future medicinal chemistry optimization. However, its high molecular weight and polarity indicate that direct oral bioavailability is unlikely without formulation or structural optimization, a limitation common to galloylated polyphenols.

Proanthocyanidin A-6 was chosen as a contrasting chemotype, demonstrating high intestinal permeability (Caco-2) and favorable metabolic safety. Its limitations included a higher risk of efflux and an elevated Ames score. As with other dimeric catechins, these properties suggest that further optimization or non-oral delivery strategies would be required for translational development.

This multi-criteria filtration, integrating initial mechanistic affinity with pharmacokinetic and safety predictions, robustly justified the selection of these three dimers for the subsequent, more computationally intensive MD and thermodynamic analysis, which is necessary to confirm their viability as molecular leads (For details, see: Table S2 and for Radar charts, see: Figures S1–S3, Supplementary Materials).

It is important to emphasize that the ADMET analysis presented here is not intended to imply conventional oral drug-likeness for the selected dimeric catechins. The high molecular weight, extensive hydrogen-bonding capacity, and elevated topological polar surface area of these compounds represent well-recognized challenges for systemic bioavailability. Rather than serving as a pass-fail filter, the ADMET profiling was used as a comparative and diagnostic framework to distinguish relative liabilities and advantages among structurally related polyphenolic scaffolds. In the context of antiviral development, such compounds are more realistically positioned as candidates for alternative delivery strategies (e.g., topical or inhaled administration), formulation-assisted approaches, or as privileged lead scaffolds for further medicinal chemistry optimization aimed at improving permeability and pharmacokinetic behavior.

It is important to emphasize that docking scores were not interpreted as quantitative measures of binding free energy, kinetic stability, or residence time. Molecular docking was used strictly as an initial, qualitative filtering tool to identify ligands with favorable geometric complementarity and interaction patterns within the fusion-regulatory pocket. Given the known limitations of docking scoring functions, all conclusions regarding stabilization, energetic favorability, and kinetic persistence were derived exclusively from post-docking analyses, including long-timescale molecular dynamics simulations, Free Energy Landscape (FEL) profiling, and MM/GBSA binding free energy estimations.

### 2.2. Molecular Dynamics Simulation and Trajectory Analysis

**Figure 4:**
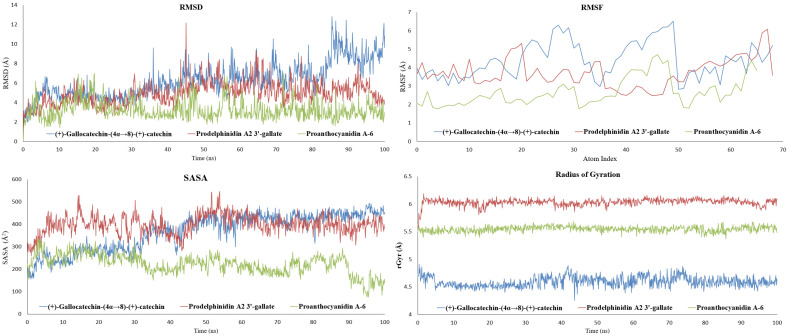
MD Simulation Metrics for HMPV F Protein Complexes over 100 ns. The composite figure displays the dynamic stability and structural metrics, RMSD plots (**Top Left**), RMSF plots (**Top Right**), SASA (**Bottom Left**) and rGyr (**Bottom Right**) for the three dimeric catechin leads: (+)-gallocatechin-(4α→8)-(+)-catechin (blue), prodelphinidin A2 3′-gallate (red), and proanthocyanidin A-6 (green).

**Figure 5:**
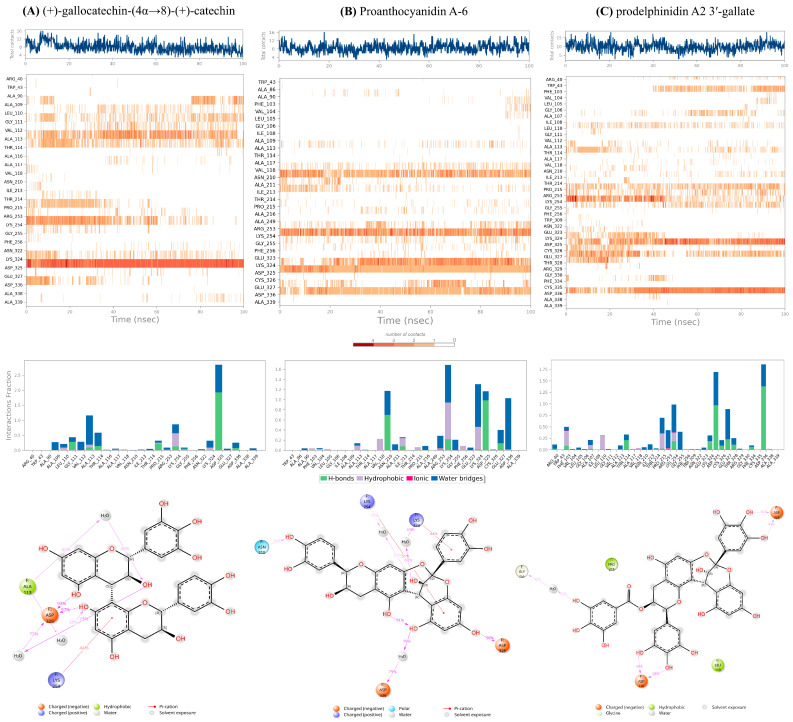
Detailed Protein-Ligand Interaction Analysis for Catechin Dimer Leads. (**A**) (+)-gallocatechin-(4α→8)-(+)-catechin, (**B**) proanthocyanidin A-6 and (**C**) prodelphinidin A2 3′-gallate, consist of: (**Top**) Interaction Timeline; (**Center**), Interaction Histogram; and (**Bottom**), Final 2D Ligand-Protein Contact Maps.

To validate the stability of the docking poses and evaluate the persistence of key inhibitory interactions, all-atom MD simulations of 100 ns were performed on the three selected HMPV F protein-ligand complexes. Analysis of the dynamic trajectories focused on RMSD and RMSF to assess global stability and local flexibility, respectively. The results are summarized graphically in Figure 4, and the protein-ligand contact analysis is shown in Figure 5 (For detailed Schrodinger Suite 2024 reports, See Section S4, Supplementary Materials).

Importantly, all three protein-ligand systems exhibited clear trajectory convergence within the simulated timescale. RMSD profiles for the protein backbone reached stable plateaus after initial equilibration, and RMSF patterns remained consistent across the remainder of the simulation, indicating stable sampling of local flexibility. Protein-ligand contact timelines further demonstrated persistent interaction networks rather than transient or drifting binding modes. These convergence features support the use of the analyzed trajectory segments for comparative assessment of relative stabilization behavior.

#### 2.2.1. Proanthocyanidin A-6 as the Most Stabilizing Complex

The complex with proanthocyanidin A-6 exhibited the highest overall stability throughout the simulation. The RMSD of the protein-ligand complex showed excellent convergence, settling rapidly and fluctuating consistently between 1.6 Å and 3.2 Å for the latter 80 ns of the trajectory. This minimal deviation indicates that the complex maintained its overall backbone conformation, and the ligand remained tightly bound within its initial pocket, confirming the predicted rigidity conferred by its A-type interflavan linkage. Correspondingly, the RMSF values remained low (1.5–3.0 Å), suggesting minimal movement in the surface loops and residues surrounding the binding site. The solvent-accessible surface area (SASA) for this complex showed a notable decrease over time (from ~325 Å^2^ to ~80–160 Å^2^), indicating that the ligand became increasingly buried and less exposed to solvent, a characteristic of robust, confined binding. Proanthocyanidin A-6 showed persistent hydrogen-bonding interactions with ASN210, LYS254, ASP325, and ASP336, suggesting that interactions with the 210–215 loop and the acidic ridge contribute prominently to the observed stabilization.

#### 2.2.2. Stable Anchoring with Flexible Sampling of Prodelphinidin A2 3′-Gallate

The prodelphinidin A2 3′-gallate complex showed moderate dynamic behavior with good stability. Its RMSD profile remained contained, stabilizing within the 5 Å to 6 Å range for the majority of the simulation, indicating adaptability without major structural collapse. This galloylated A-type dimer maintained strong and persistent hydrogen bonds with the critical acidic residues, specifically ASP325 and ASP336. Additionally, it exhibited intermittent interactions with neighboring polar and aromatic residues (THR114, ARG253, PHE334) as indicated by the contact timeline. This behavior suggests a dynamic profile where the core of the ligand remains strongly anchored to the acidic ridge, while its flexible peripheral groups (the galloyl moiety) allow it to sample and stabilize various nearby binding patches within the 110–115 and 210–215 loop regions. This balance of rigid anchoring and flexible sampling is key to its high therapeutic potential.

#### 2.2.3. Adaptability to Loop Motion of (+)-Gallocatechin-(4α→8)-(+)-Catechin

In contrast, the (+)-gallocatechin-(4α→8)-(+)-catechin complex, characterized by a flexible B-type linkage and pyrogallol rings, showed the highest RMSD fluctuations, rising to a range of 7.5 Å to 9.5 Å toward the end of the simulation. This increase was primarily attributed to the relaxation and movement of highly flexible surface loops (RMSF values of 3–5 Å) rather than global protein unfolding. Crucially, even with these larger global protein fluctuations, the ligand maintained persistent local contacts, particularly via hydrogen bonding to ASP325 and its preference for the 110–115 loop region. The flexible nature of this ligand allows it to adapt its orientation as the protein surface “breathes,” preserving stabilizing local contacts despite high global RMSD.

The MD results therefore validate a direct Structure-Dynamics Relationship (SDR) among the leads: the rigid A-type linkage of proanthocyanidin A-6 promotes the most compact and stable structural lock, while the galloylation of prodelphinidin A2 3′-gallate provides persistent anchoring with localized dynamic flexibility, and the B-type linkage of (+)-gallocatechin-(4α→8)-(+)-catechin enables accommodation of larger protein loop motions. The next step is to quantify the thermodynamic consequences of these distinct dynamic profiles.

#### 2.2.4. Ligand Atomic Fluctuations (L-RMSF)

To dissect the influence of specific chemical moieties on the binding dynamics, the Ligand Root Mean Square Fluctuation (L-RMSF) was analyzed over the 100 ns trajectory (Section S4, Supplementary Materials). The L-RMSF plots reveal that for prodelphinidin A2 3′-gallate, the lowest atomic fluctuation occurred within the two core pyran rings and the A-B fused rings, confirming these regions are tightly anchored in the binding pocket. In contrast, the terminal galloyl group exhibited marginally higher L-RMSF values. This differential movement confirms that the electrostatic-rich galloyl moiety retains sufficient local flexibility to optimize its H-bond and π-π stacking arrangements with surrounding residues (PHE256, ASP325), a movement that facilitates the strong electrostatic contribution observed in the final MM/GBSA calculation. This evidence of local, functional flexibility further reinforces its selection as the primary lead.

### 2.3. Free Energy Landscape (FEL) Analysis

**Figure 6:**
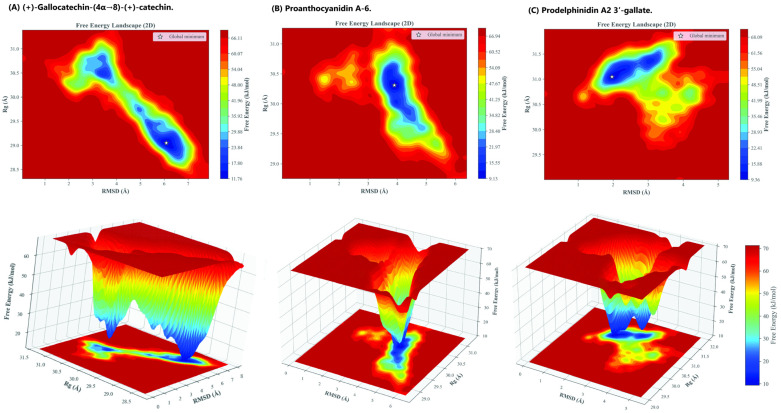
FEL Analysis of HMPV F Glycoprotein-Ligand Complexes. The 2D and 3D maps project the Gibbs free energy (Δ*G* in kJ/mol) as a function of two collective variables: RMSD, (*x*-axis) and R_g_, (*y*-axis). The global minimum for each complex (indicated by the white star) represents the most thermodynamically favored binding conformation sampled during the 100 ns simulation. (**A**) (+)-Gallocatechin-(4α→8)-(+)-catechin. (**B**) Proanthocyanidin A-6. (**C**) Prodelphinidin A2 3′-gallate.

To obtain a deeper thermodynamic understanding of how each ligand influences the conformational preference and structural ensemble of the HMPV F glycoprotein, a Free Energy Landscape (FEL) analysis was performed on the converged 100 ns MD trajectories. The Gibbs free energy (Δ*G*) was projected as a function of the two primary collective variables: the Root Mean Square Deviation (RMSD) and the Radius of Gyration (*R_g_*) of the protein backbone. The resulting 2D and 3D FEL maps, shown in Figure 6, reveal the favored, low-energy conformational states stabilized by each dimeric catechin.

The FEL analysis provides qualitative, trajectory-specific insight into conformational restriction within the sampled simulations imposed by the ligands:

Prodelphinidin A2 3′-gallate was the most effective in stabilizing a single, highly constrained prefusion state. Its FEL profile displayed a single, sharp global minimum at *RMSD* = 1.968 Å and *R_g_* = 31.047 Å (Δ*G* = 9.357 kJ/mol). This narrow, deep energy basin within the sampled trajectory suggests minimal structural rearrangement throughout the simulation, confirming strong stabilization of the prefusion backbone conformation. The strong π-π and hydrogen-bonding interactions provided by the galloyl substitution act as a highly effective anchor, maximizing the energy required for the protein to explore alternative, potentially fusogenic conformations.

In comparison, the Proanthocyanidin A-6 complex also showed strong conformational constraint, yielding a relatively compact and well-defined global minimum at Δ*G* = 9.126 kJ/mol (*RMSD* = 3.899 Å, *R_g_* = 30.310 Å). While numerically superior in the FEL minimum value (ΔG), this single, deep well signifies rigidity and structural compactness, aligning with the observed low RMSF values.

In contrast, the (+)-gallocatechin-(4α→8)-(+)-catechin complex exhibited a broader, shallower energy well with multiple distinct low-energy regions. The global minimum was observed at a higher free energy value (Δ*G* = 11.764 kJ/mol) and higher RMSD (6.085 Å, *R_g_* = 29.050 Å). The presence of multiple minima confirms that the ligand’s flexible B-type interflavan linkage allows the protein to explore a wider range of prefusion-like conformational substates, suggesting moderate stabilization rather than complete structural constraint.

The FEL profiles corroborate the dynamic observations, showing that the most potent compounds induce the greatest energetic restraint. The structural confinement dictated by Prodelphinidin A2 3′-gallate provides the essential kinetic stabilization for a fusion-locking mechanism.

### 2.4. Thermodynamic Stability Analysis (MM/GBSA and MM/PBSA)

To translate the dynamic behavior observed in the 100 ns MD trajectories into comparative energetic trends, the MM/GBSA and MM/PBSA end-point binding free energy methods were employed. Unlike docking scores, MM/GBSA estimates provide post-MD energetic evaluations based on ensemble-averaged conformations and therefore offer a more appropriate proxy for comparative binding energetics. These calculations were performed on representative snapshots extracted from the most stable, converged regions of each complex’s trajectory. This dual approach provides a robust thermodynamic comparison, where the MM/GBSA method (using the Generalized Born continuum solvation model) is often superior for ranking relative binding affinities in large molecular systems. The final ΔG_binding_ values and a breakdown of the energetic contributions were calculated and are summarized in Table 2.

A clear, convergent trend in the overall thermodynamic stability was observed, reinforcing the structural insights from the dynamic analysis (RMSD/RMSF):

Δ*G_MMGBSA_*: (+)-gallocatechin-(4α→8)-(+)-catechin > Proanthocyanidin A-6

Prodelphinidin A2 3′-gallate demonstrated the most favorable overall binding free energy (Δ*G_MMGBSA_* = −42.43 kcal/mol; Δ*G_MMGBSA_* = −6.24 kcal/mol). This thermodynamic superiority is attributed to a highly favorable balance of two core components:

#### 2.4.1. Electrostatic and Van Der Waals Contributions

This complex achieved strong van der Waals forces (Δ*E_vdw_* = −74.78 kcal/mol) and the most favorable electrostatic term (Δ*E_elec_* = −32.48 kcal/mol). This high Δ*E_elec_* is directly linked to the presence of the galloyl moiety, which maximizes hydrogen bonding and aromatic π-π stacking interactions with polar/acidic residues within the binding site.

#### 2.4.2. Solvation Penalty Management

Despite its high number of hydroxyl groups, the large Δ*E_MM_* (Molecular Mechanics component) significantly overcame the associated polar solvation penalty (Δ*G_polar solvation_* = +68.58 kcal/mol).

This data confirms that the balanced dynamic profile observed for prodelphinidin A2 3′-gallate (strong residue-level anchoring coupled with localized flexibility) translates to the most energetically rewarding binding mode, thus positioning it as the primary lead.

(+)-gallocatechin-(4α→8)-(+)-catechin showed moderate thermodynamic favorability (Δ*G_MMGBSA_* = −33.38 kcal/mol; Δ*G_MMGBSA_* = −15.65 kcal/mol). The B-type dimer provided a balanced contribution from both non-polar (Δ*E_vdw_*) and electrostatic (Δ*E_elec_* = −25.90 kcal/mol) sources. Its flexible linkage permits dynamic adaptation to loop motions, which prevents a deeper, more stabilized van der Waals confinement but ensures persistent polar contacts, resulting in an intermediate energy profile.

Proanthocyanidin A-6 exhibited the least favorable energetics (Δ*G_MMGBSA_* = −15.08 kcal/mol), despite being the most structurally rigid complex in the MD trajectory (Section 3.2). This apparent discrepancy is mechanistically significant: the compound’s stiff A-type linkage and lack of a galloyl group led to a restricted conformational accommodation within the binding pocket. While this rigidity resulted in minimal RMSD fluctuations, it prevented the formation of necessary deep or extended electrostatic and non-polar contacts. Consequently, the beneficial van der Waals energy achieved was severely counterbalanced by an extremely large polar solvation penalty (Δ*G_polar solvation_* ranging from +73.84 to +80.01 kcal/mol), rendering the overall binding affinity poor.

The observation that the most structurally rigid complex (Proanthocyanidin A-6) exhibited the poorest binding free energy (Δ*G_MMGBSA_*) highlights the necessity of dynamic solvation analysis over static docking snapshots. This stability paradox underscores that kinetic stabilization, rather than mere conformational rigidity, is the true determinant of a potent fusion-locking agent, a distinction only achievable through the high-resolution thermodynamic integration employed in this study. The highest quality lead, prodelphinidin A2 3′-gallate, succeeded by achieving the optimal balance between strong galloylation-driven electrostatic anchoring and sufficient flexibility to minimize the unfavorable desolvation cost.

### 2.5. Mechanistic and Structure-Dynamics Implications (Fusion-Locking)

For clarity, the mechanistic terms used throughout this study are defined explicitly. “Anchoring” refers to localized, persistent residue-level interactions between a ligand and specific functional regions of the HMPV F protein, particularly stable hydrogen bonding and electrostatic contacts with the Asp325-Asp336 acidic ridge. “Stabilization” denotes the resulting global energetic and dynamic effect on the prefusion F protein, reflected by reduced conformational flexibility, constrained free energy landscapes, and favorable binding free energies. “Fusion-locking” (or “locking”) describes the functional consequence of anchoring-driven stabilization, wherein the conformational rearrangements required for the irreversible prefusion-to-postfusion transition are comparatively hindered within the simulated timescale, thereby preventing membrane fusion.

Although the Asp325-Asp336 acidic ridge emerges as a recurrent interaction site in this study, we do not claim that this region is uniquely stabilized relative to all other regions of the HMPV F protein. Rather, its mechanistic relevance is inferred from convergent indicators, including persistent ligand contact frequencies, low local RMSF values, ligand atomic fluctuation localization, and its consistent involvement across docking, MD interaction timelines, FEL minima, and MM/GBSA energetic contributions. Within the scope of the present analyses, these combined observations identify the Asp325-Asp336 region as a primary stabilization hotspot rather than as an exclusively stabilized site.

The integrated computational data from docking, MD simulation, and MM/GBSA binding free energy calculations strongly converge to support a distinct mechanism of action for the dimeric catechins: stabilization of the HMPV F protein in its metastable prefusion conformation, functioning as a fusion-locking agent.

The HMPV F glycoprotein must undergo a major, irreversible conformational transition (from prefusion to postfusion) to facilitate membrane fusion and viral entry. This transition is mediated by the flexibility of specific surface elements, most notably the acidic ridge (Asp325-Asp336) and adjacent loop regions (110–115 and 210–215), which are critical for inter-protomer interactions and triggering.

The potent leads identified, particularly prodelphinidin A2 3′-gallate, consistently demonstrated persistent anchoring interactions with these regions across the 100 ns trajectory. The mechanistic role is supported by the following structural findings:

#### 2.5.1. Targeted Anchoring

All high-scoring compounds maintained persistent hydrogen-bonding and ionic contacts with Asp325 and Asp336. The final 2D Ligand-Protein Contact Maps (Figure 5, Bottom) explicitly confirm that the hydroxyl groups of the core catechin rings and the galloyl moiety form strong, direct contacts (>70 persistence) with the side chains of ASP325 and ASP336. In the case of prodelphinidin A2 3′-gallate, the galloyl moiety provided the necessary electrostatic complementarity to maximize Δ*E_elec_* (Section 3.3) and maintain this anchoring lock.

#### 2.5.2. Restricted Flexibility

The localized nature of the binding, confirmed by low RMSF in the direct binding site, physically constrains the necessary movement of the 110–115 and 210–215 loops. This inhibition of loop breathing raises the energy barrier for the conformational shift, resulting in effective fusion-locking of the prefusion state.

#### 2.5.3. Structural Chemotype Influence

The quantitative comparison of the dimeric structures revealed a critical Structure-Dynamics relationship: the flexible B-type linkage of (+)-gallocatechin-(4α→8)-(+)-catechin allowed it to dynamically accommodate protein fluctuations (high RMSD), while the rigid A-type scaffold of proanthocyanidin A-6 was too stiff, leading to a high desolvation penalty (poor Δ*G*). Prodelphinidin A2 3′-gallate achieved the optimal balance, using its galloyl group to provide thermodynamic stability while retaining sufficient localized flexibility to minimize energetic cost.

The stabilization of the metastable prefusion conformation is a recognized and highly desirable strategy for antiviral development against Class I viral fusion proteins (such as those found in RSV and Influenza). This robust, integrated MD/MM/GBSA analysis thus provides a strong computational proof-of-concept, concluding that prodelphinidin A2 3′-gallate is the most potent stabilizing agent among the leads identified. We acknowledge that the use of single-replica simulations represents a limitation of the present study, and that future investigations employing multiple independent replicas or enhanced sampling techniques would be valuable to further assess the robustness and long-timescale persistence of the observed stabilization effects.

### 2.6. Post-MD Ramachandran Plot Analysis

To ensure the structural integrity of the HMPV F protein was preserved during the 100 ns MD simulations and that the observed binding poses were stereochemically valid, a Ramachandran plot analysis was conducted on the final trajectory frame of each protein-ligand complex. This step is essential for validating the quality of computational models prior to experimental advancement.

**Figure 7:**
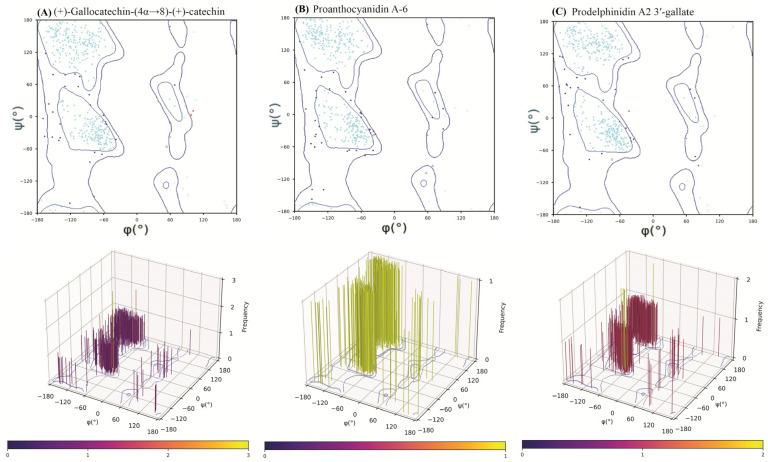
Post-MD Ramachandran 2D and 3D Plot Analysis of the HMPV F Protein Backbone Conformation. 2D plots: Cyan, blue and red (dots/triangles) represent torsion angles of favored, allowed and disallowed regions respectively; dot represents residues other than glycine and triangles represents glycine.

Across all three complexes, the protein backbone dihedral angles (*ϕ* and *ψ*) maintained excellent stereochemical quality (Figure 7). The percentage of residues residing in the most favored regions were consistently high: prodelphinidin A2 3′-gallate (92.52%), (+)-gallocatechin-(4α→8)-(+)-catechin (92.82%), and proanthocyanidin A-6 (90.61%). Similarly, the percentage of residues in the disallowed regions remained minimal (0.46–1.40%).

The high percentage of favored residues, well above the threshold typically used for high-quality X-ray crystal structures (>90%), confirms that the binding of the large catechin dimers and the subsequent loop movements during the dynamic simulation did not induce any significant, irreversible backbone strain or stereochemical violations in the HMPV F protein. The results from the 3D torsional frequency plots (Figure 7) further reinforced these findings, showing densely clustered populations in canonical regions, with prodelphinidin A2 3′-gallate exhibiting the most distinctly defined clustering, signifying optimal conformational convergence during dynamic equilibration. This validation step confirms that the calculated dynamic and thermodynamic data are based on chemically and sterically plausible final receptor conformations.

## 3. Materials and Methods

### 3.1. Target and Ligand Preparation

The three-dimensional crystal structure of the HMPV F glycoprotein stabilized in its pre-fusion conformation (PDB ID: 5WB0) was retrieved from the Research Collaboratory for Structural Bioinformatics (RCSB) Protein Data Bank. Prior to molecular docking, the protein structure was prepared by removing non-essential co-crystallized water molecules, extraneous ions, and the Triz-BME stabilizer molecule to ensure a clean receptor surface. Hydrogen atoms were added, and protonation states for histidine residues were assigned according to physiological *pH* (7.4). For all catechin-based ligands, protonation states were assigned assuming physiological *pH* (7.4). Phenolic hydroxyl groups present in catechin and galloyl moieties were modeled in their neutral (protonated) form, consistent with their reported *pKa* values (>8.5–9.5) and experimental evidence indicating that these groups remain largely unionized under physiological conditions. This treatment preserves the hydrogen-bond donor capability of the polyphenolic scaffold, which is essential for realistic modeling of the extensive hydrogen-bonding and electrostatic complementarity observed between galloylated catechins and the Asp325-Asp336 acidic ridge of the HMPV F protein. No artificial deprotonation states were imposed, ensuring chemically and biologically relevant ligand representations.

The ligand library, comprising a total of 210 phytochemicals reported from *C. sinensis*, was sourced from the Indian Medicinal Plants, Phytochemistry, and Therapeutics (IMPPAT, https://cb.imsc.res.in/imppat/, accessed on 23 December 2024) database [24,25]. The structures were optimized using the Universal Force Field (UFF, Avogadro 1.2.0) and converted to PDBQT format for subsequent docking simulations. EGCG, a known HMPV entry inhibitor and major tea constituent, was included in the library and served as the benchmark control reference compound for affinity comparisons.

### 3.2. Docking-Based Virtual Screening and ADMET Filtration

Initial virtual screening was performed using the CBDoCK-2 web server [26], which employs the AutoDock Vina (version 1.2.0) force field for efficient rigid-receptor molecular docking. The binding pocket of the HMPV F protein was defined by generating a grid box centered on the known fusion-regulatory regions, specifically the Asp325-Asp336 acidic ridge and the adjacent loop areas (110–115 and 210–215), which are implicated in conformational flexibility and membrane fusion triggering.

The 210 phytochemicals were ranked based on their calculated binding affinity (kcal/mol). Compounds exhibiting a better score than the reference compound (EGCG, −8.6 kcal/mol) were prioritized for further analysis. This initial selection was then subjected to ADMET (Absorption, Distribution, Metabolism, Excretion, and Toxicity) profiling using the ProTox 3.0 server (https://tox.charite.de/protox3/, accessed on 20 February 2025) [27]. This step served as a crucial computational filtration layer to exclude compounds with poor pharmacokinetic properties or high predicted toxicity risks (mutagenicity, hERG cardiotoxicity). Based on the combination of superior theoretical binding stabilization and optimal ADMET profiles (favorable solubility, low efflux risk, and minimal CYP450 interaction), the top three dimeric leads (+)-gallocatechin-(4α→8)-(+)-catechin, prodelphinidin A2 3′-gallate, and proanthocyanidin A-6 were selected for in-depth dynamic simulation (Table S1, Supplementary Materials). For each of the top-ranked ligands, docking poses were not selected solely based on binding affinity scores. Instead, a multi-criteria pose selection strategy was applied prior to MD simulations. The final poses were chosen based on (i) favorable docking energy relative to EGCG, (ii) correct spatial orientation toward the functionally critical Asp325-Asp336 acidic ridge and adjacent loop regions (110–115 and 210–215), (iii) formation of key hydrogen-bonding and aromatic interactions with residues implicated in fusion triggering, and (iv) absence of steric clashes or strained ligand conformations upon visual inspection. When multiple poses met these criteria, the pose exhibiting the highest number of persistent polar contacts with Asp325 and Asp336 was selected for downstream MD simulations.

### 3.3. Molecular Dynamics (MD) Simulation Setup

The stability and dynamic behavior of the final three HMPV F protein-ligand complexes were investigated using all-atom MD simulations. Simulations were carried out using the Desmond module within the Schrodinger Suite 2024 (v1.6) [28]. The purpose was to model the system under near-physiological conditions to assess the stability and persistence of the predicted binding interactions over time, a critical step in verifying the computational hypothesis.

The Preparation and Equilibration phases followed a strict protocol to ensure system balance. Each complex (protein and ligand) was first positioned within an orthorhombic simulation box. The system was then explicitly solvated with TIP3P water molecules and neutralized by adding appropriate counterions (Na^+^ or C^−^) to establish a physiological salt concentration of 0.15 M NaCl. The industry-standard OPLS3e force field [29], widely recognized for its robustness and accuracy in modeling protein-ligand systems, was applied to the entire system (protein, ligand, and solvent). The OPLS3e force field was specifically selected due to its enhanced parametrization for drug-like and natural product chemical space, including aromatic polyphenols and highly functionalized oxygen-rich scaffolds. OPLS3e incorporates refined torsional parameters, improved treatment of hydrogen bonding, and accurate partial charge assignment, which are critical for capturing the complex electrostatic and dispersion interactions governing catechin-protein binding. Its demonstrated performance in modeling protein-polyphenol systems and large flexible ligands makes it particularly suitable for the present study, where galloylated dimeric catechins engage in multi-point hydrogen bonding and π-interactions within a dynamic protein environment. The system underwent a relaxation protocol, which began with restrained energy minimization to alleviate initial steric clashes, followed by a multi-step thermal and pressure equilibration phase using the NPT ensemble (300 K, 1 bar) controlled by the Langevin thermostat and barostat [30,31].

The production runs were conducted under the NPT ensemble for a total duration of 100 ns for each of the three complexes to ensure sufficient sampling of the conformational space. The SHAKE algorithm [32] was employed to constrain bond lengths involving hydrogen atoms, allowing for a larger time step, and long-range electrostatic interactions were efficiently handled using the Particle Mesh Ewald (PME) method [33]. The resulting trajectories were analyzed for key structural metrics including Root Mean Square Deviation (RMSD), Root Mean Square Fluctuation (RMSF), and specific protein-ligand contact analysis to determine stable binding poses and persistent interactions over the full 100 ns period.

Single-replica 100 ns MD simulations were employed as a comparative and mechanistic framework to evaluate relative stability trends among closely related catechin dimers. This simulation length was selected based on prior studies of viral fusion proteins, where 100 ns trajectories are sufficient to assess local loop flexibility, protein-ligand contact persistence, and convergence of RMSD/RMSF metrics. Convergence was explicitly evaluated by monitoring RMSD plateaus, RMSF consistency across structured and flexible regions, and stabilization of key protein–ligand interaction patterns. While multiple independent replicas can further enhance statistical robustness, the present approach was designed to provide internally consistent, relative comparisons rather than absolute kinetic rate estimations.

### 3.4. Free Energy Landscape (FEL) Generation Protocol

To gain deeper insight into the conformational stability and kinetic barriers associated with ligand binding, the Free Energy Landscape (FEL) analysis was performed using the post-production MD trajectories. The FEL provides a detailed map of the thermodynamically accessible conformational states, which is essential for characterizing a stable fusion-locking mechanism [34,35]. Because the FEL projections are derived from single trajectories and a limited set of collective variables, they represent qualitative descriptions of conformational restriction within the sampled conformational space rather than definitive reconstructions of the underlying free energy surface.

The Gibbs free energy (Δ*G*) was calculated via the standard Boltzmann inversion method, based on the probability distribution of sampled states:

$$∆G\left(X\right)=-k_{B}T\cdot ln\frac{P(X)}{P_{max}}$$

where *k_B_* is the Boltzmann constant, *T* is the simulation temperature (300 K), *P(X)* is the probability density function of the system at state *X*, and *P_max_* is the probability of the most populated state, which is set as the energy reference. The conformational space (*X*) was defined by projecting the protein trajectory onto two primary collective variables (CVs): the RMSD and the *R_g_* of the protein backbone. The resulting 2D maps identify low-energy basins (Δ*G_min_*) which correspond to the most stable conformations achieved by the protein-ligand complex during the 100 ns simulation.

### 3.5. Binding Free Energy Calculations (MM/GBSA and MM/PBSA)

To quantify the thermodynamic driving forces of ligand binding, the Molecular Mechanics/Generalized Born Surface Area (MM/GBSA) and Molecular Mechanics/Poisson-Boltzmann Surface Area (MM/PBSA) methods were applied to snapshots extracted from the stable regions of the 100 ns MD trajectories [36,37]. These end-point calculations provide relative energetic estimates of ligand binding by decomposing molecular mechanics (Δ*E_MM_*) and solvation (Δ*G_solvation_*) contributions and are primarily intended for comparative ranking within a consistent simulation framework rather than for quantitative determination of absolute binding free energies. MM/GBSA and MM/PBSA calculations were performed as end-point estimates using a limited number of representative snapshots extracted from converged regions of the MD trajectories. These calculations were intended for relative comparison of ligand stability within the same simulation protocol and were not designed to yield absolute binding free energies. Given the sparse sampling and absence of replica averaging, the resulting values should be interpreted qualitatively rather than as thermodynamically rigorous estimates.

The Δ*G_binding_* for the MM/GBSA approach, as calculated by the UNI-GBSA workflow, follows the decomposition:

$${∆G}_{binding}=G_{complex}-G_{receptor}-G_{ligand}$$

where each component *G* is represented as:

$$G=E_{MM}+G_{solvation}$$

The total binding free energy is thus decomposed into the molecular-mechanical energy term (Δ*E_MM_*) and the solvation free energy term (Δ*G_solvation_*):

$${∆G}_{binding}={∆E}_{internal}+{∆E}_{vdw}+{∆E}_{electrostatic}+{∆G}_{polar}+{∆G}_{nonpolar}$$

Calculations were performed using the UNI-GBSA software (Avogadro 1.2.0) platform [38]. Snapshots were collected from the converged and stable regions of the MD production phase to ensure that the calculated energies represented the most populated and biologically relevant binding modes. The final reported Δ*G_binding_* values represent the average calculated over three distinct, stable time points for each complex, providing robust statistical convergence and allowing for a direct comparison of thermodynamic stability among the three catechin dimers. Post-MD structural integrity was assessed using the Ramachandran plot analysis via the Ramplot server (https://www.ramplot.in/, accessed on 17 September 2025) [39].

## 4. Conclusions

This study presents a comparative and mechanistically informed computational framework for the systematic identification and computational evaluation of relative stabilization mechanisms of HMPV fusion inhibitors from *Camellia sinensis*. By integrating 100 ns MD simulations with Free Energy Landscape (FEL) and MM/GBSA analysis, we moved beyond static docking to characterize the kinetic persistence of natural dimeric catechins within the Fusion (F) glycoprotein. The analysis robustly identified prodelphinidin A2 3′-gallate as the primary lead. While other dimers showed structural rigidity, prodelphinidin A2 3′-gallate achieved the optimal balance between galloylation-driven electrostatic anchoring and dynamic flexibility, resulting in a superior binding free energy (Δ*G_MMGBSA_* = −42.43 kcal/mol). This superiority is rooted in the compound’s efficient management of the polar solvation penalty, a critical determinant for bioactive natural products. Mechanistically, our findings provide a detailed structural hypothesis for anchoring interactions involving the Asp325-Asp336 acidic ridge that contribute to fusion-locking of the prefusion conformation. This integrated workflow establishes a reproducible, evidence-based roadmap for the subsequent experimental synthesis and biophysical validation of these natural product-inspired inhibitors. Future studies incorporating multiple independent replicas, denser energetic sampling, and enhanced sampling approaches would be valuable to further quantify binding thermodynamics and refine the free energy landscape of ligand-mediated stabilization.

## Figures and Tables

**Table 1:** Predicted ADMET and Physicochemical Properties of the Three Selected Dimeric Catechins.

	MW	Vol	TPSA	logS	logD	logP	MDCK	PAMPA	hia	hERG	Ames
(+)-gallocatechin-(4α→8)-(+)-catechin	594.140	558.733	240.990	−2.898	1.465	1.010	−4.883	0.670	0.009	0.158	0.259
Prodelphinidin A2 3-gallate	760.130	696.097	316.980	−4.153	0.421	0.608	−4.910	0.941	0.000	0.069	0.740
Proanthocyanidin A-6	576.130	541.386	209.760	−3.238	1.650	1.468	−4.875	0.546	0.000	0.110	0.712

**Table 2:** Binding Free Energy Results (kcal/mol) for HMPV F Protein-Ligand Complexes.

Compound	MM/GBSA (kcal/mol)	MM/PBSA (kcal/mol)
(+)-Gallocatechin-(4α→8)-(+)-catechin	−33.17	−10.92
Proanthocyanidin A-6	−25.94	+18.01
Prodelphinidin A2 3′-gallate	−42.43	−6.24

## Data Availability

All software used in this work is open source or licensed as stated in the methodology. All raw data generated in this study, including ligand structures, minimized protein-ligand coordinates (PDB), MD analysis time-series data (RMSD, RMSF, rGyr), MM/GBSA component values, and the full docking/ADMET screening tables, are available on the following GitHub repository: https://github.com/tusharpawar49/HMPV_FusionLocking_Dimeric_Catechins.

## Supplementary Materials

The following supporting information can be downloaded at https://www.mdpi.com/article/10.3390/molecules31050821/s1, Section S1. Top 17 Compounds Selected Based on Docking Score: Table S1: Docking results obtained from the phytoconstituents under study. Section S2. ADMET: Table S2: Detailed ADMET properties of (+)-gallocatechin-(4α→8)-(+)-catechin, Prodelphinidin A2 3-gallate and Proanthocyanidin A-6. Figure S1: Radar Chart of (+)-gallocatechin-(4α→8)-(+)-catechin. Figure S2: Radar Chart of Prodelphinidin A2 3-gallate. Figure S3: Radar Chart of Proanthocyanidin A-6. Section S3. Molecular Docking: Figure S4: Molecular Docking Visualization of (+)-gallocatechin-(4α→8)-(+)-catechin. Figure S5: The predicted binding modes and key residue contacts for (+)-gallocatechin-(4α→8)-(+)-catechin. Figure S6: Molecular Docking Visualization of Prodelphinidin A2 3-gallate. Figure S7: The predicted binding modes and key residue contacts for Prodelphinidin A2 3-gallate. Figure S8: Molecular Docking Visualization of Proanthocyanidin A-6. Figure S9: The predicted binding modes and key residue contacts for Proanthocyanidin A-6. Section S4. Molecular Dynamics (MD) Simulation: Schrodinger Suite 2024 (v1.6) reports of (+)-gallocatechin-(4α→8)-(+)-catechin, proanthocyanidin A-6, and prodelphinidin A2 3′-gallate.
