# Supplementary material for: Galloylation-Driven Anchoring of the Asp325-Asp336 Ridge: The Molecular Logic Behind the Superior Kinetic Stabilization of HMPV Fusion Protein by Green Tea Dimeric Catechins
Source: Molecules. 2026 Feb 28;31(5):821. doi: 10.3390/molecules31050821 (PMC12986219; doi:10.3390/molecules31050821)
Supplement: Supplementary file 1 [file molecules-31-00821-s001.zip › Supporting Information.pdf]

Supplementary File

Galloylation-Driven Anchoring of the Asp325–Asp336 Ridge: The Molecular Logic Behind the Superior Kinetic Stabilization of HMPV Fusion Protein by Green Tea Dimeric Catechins

Shrikant S. Nilewar [1], Santosh S. Chobe [2], Amruta D. Gurav [3], Salman B. Kureshi [3], Srushti B. Palande [3], Jesica Escobar-Cabrera [4], Fabiola Hernandez-Rosas [4,5], and Tushar Janardan Pawar [6,7,*]

1 Department of Pharmaceutical Chemistry, Maliba Pharmacy College, Uka Tarsadia University, Bardoli 394350,
Gujrat, India; shrinilewar@gmail.com
2 Department of Chemistry, M.G.V.'s Loknete Vyankatrao Hiray, Arts, Science and Commerce College,
Panchavati, Nashik 422003, Maharashtra, India; chobess222@gmail.com
3 School of Pharmaceutical Science, Sandip University, Nashik 422213, Maharashtra, India;
amrutagurav70@gmail.com (A.D.G.); salmankureshi216@gmail.com (S.B.K.);
srushti.palande1704@gmail.com (S.B.P.)
4 Facultad de Quimica, Universidad Autonoma de Queretaro, Queretaro 76010, Mexico;
jesica.escobar@uaq.mx (J.E.-C.); fabiola.hernandezro@anahuac.mx (F.H.-R.)
5 Centro de investigacion, Universidad Anahuac Queretaro, Circuito Universidades I, Fraccion 2 S/N, Zibata,
El Marques, Queretaro 76246, Mexico
6 Direccion de Mecatronica, Universidad Politecnica de Queretaro, Carretera Estatal 420 S/N, El Rosario,
Queretaro 76240, Mexico
7 Escuela de Ingenieria Quimica, Universidad Anahuac Queretaro, Circuito Universidades I, Fraccion 2 S/N,
Zibata, El Marques, Queretaro 76246, Mexico
* Correspondence: tushar.pawar@anahuac.mx

S1. Top 17 Compounds Selected Based on Docking Score

Table S1: Docking results obtained from the phytoconstituents under study.

Sr. No.	IMPAAT Id	_Phytochemical Name	Docking Score
1	IMPHY010264	(+)-Gallocatechin-(4α→8)-(+)-catechin	-10.0
2	IMPHY000833	Theasinensin C	-9.9
3	IMPHY013513	Theaflavin-3'-O-gallate	-9.7
4	IMPHY000786	Theasinensin F	-9.4
5	IMPHY002722	Prodelphinidin A2 3'-gallate	-9.4
6	IMPHY005824	Benzoic acid, 3,4,5-trimethoxy-, diester with 1,8-*bis*(3,4-dihydro-3,5,7-trihydroxy-2*H*-1-benzopyran-2-yl)-3,4,6-trihydroxy-5*H*-benzocyclohepten-5-one	-9.4
7	IMPHY000697	[(*2R*,*3R*)-2-[8-[5,7-dihydroxy-3-(3,4,5-trihydroxybenzoyl)oxy-3,4-dihydro-2*H*-chromen-2-yl]-3a,5,6-trihydroxy-3-oxo-8bH-cyclopenta[b][1]benzofuran-1-yl]-5,7-dihydroxy-3,4-dihydro-2*H*-chromen-3-yl] 3,4,5-trihydroxybenzoate	-9.3
8	IMPHY001777	5*H*-Benzocyclohepten-5-one, 1,8-bis((*2R*,*3R*)-3,5,7-trihydroxy-2*H*-1-benzopyran-2-yl)-3,4,6-trihydroxy-	-9.3
9	IMPHY000790	Proanthocyanidin A-6	-9.2
10	IMPHY005089	Theaflavin monogallates	-9.2
11	IMPHY001089	Theasinensin A	-9.0
12	IMPHY004845	Gallocatechin-(4α→8)-epigallocatechin	-9.0
13	IMPHY006999	Strictinin	-9.0
14	IMPHY004847	Epigallocatechin-(4β→8)-epicatechin-3-*O*-gallate ester	-8.9
15	IMPHY014842	Stigmasterol	-8.9
16	IMPHY010606	3,3'-Digalloylprocyanidin B2	-8.7
17	IMPHY011670	Epigallocatechin gallate	-8.6

S2. ADMET

Table S2: Detailed ADMET properties of (+)-gallocatechin-(4α→8)-(+)-catechin, Prodelphinidin A2 3-gallate and Proanthocyanidin A-6.

Property	(+)-gallocatechin-(4α→8)-(+)-catechin	Prodelphinidin A2 3-gallate	Proanthocyanidin A-6
MW	594.14	760.13	576.13
Vol	558.73	696.097	541.386
Dense	1.063	1.092	1.064
nHA	13	18	12
nHD	11	13	9
TPSA	240.99	316.98	209.76
nRot	3	5	2
nRing	6	8	7
MaxRing	10	20	20
nHet	13	18	12
gasa	1	1	1
QED	0.153	0.092	0.158
Synth	4.625	5.829	5.378
Fsp3	0.2	0.162	0.2
MCE-18	130	173.791	143.333
Natural Product-likeness	2.092	1.785	2.144
Lipinski	1	1	1
Pfizer	0	0	0
GSK	1	1	1
GoldenTriangle	1	1	1
logS	-2.89779841	-4.15261064	-3.23780711
logD	1.46524691	0.42113402	1.65048975
logP	1.00974024	0.60780415	1.46763211
mp	167.668789	413.261821	210.57245
bp	351.066807	578.116957	368.090097
pka_acidic	6.93599498	7.80303741	7.84262451
pka_basic	4.06565793	0.31905712	5.06234934
caco2	-7.09899165	-7.42517825	-6.98212649
MDCK	-4.88287968	-4.91006901	-4.87492647
PAMPA	0.67030668	0.94066304	0.54612333
pgp_inh	4.70E-07	1.02E-07	8.52E-06
pgp_sub	0.03818047	0.00505844	0.21493895
hia	0.00899661	0.00011826	2.32E-05

Property	(+)-gallocatechin-(4α→8)-(+)-catechin	Prodelphinidin A2 3-gallate	Proanthocyanidin A-6
f20	0.99811686	0.9997425	0.9739325
f30	0.99999853	0.9999935	0.99960674
f50	0.99999306	0.99999992	0.99995519
OATP1B1	0.99996114	0.95837826	0.9925344
OATP1B3	0.99988472	0.98692155	0.90880889
BCRP	0.09363233	0.7194885	0.0006409
BSEP	3.46E-05	0.00127815	0.51625544
BBB	0.00088382	0.00072622	1.48E-05
MRP1	0.98130441	0.99998629	0.97272146
PPB	89.3987316	88.6441966	93.7284572
logVDss	0.19989328	-0.05646246	0.18760303
Fu	17.3200945	11.326487	8.25754289
CYP1A2-inh	5.59E-15	5.68E-10	2.29E-17
CYP1A2-sub	0.0966195	6.15E-06	0.01155146
CYP2C19-inh	2.73E-11	3.18E-07	2.05E-08
CYP2C19-sub	1.39E-07	1.12E-10	1.79E-08
CYP2C9-inh	8.86E-08	0.52721131	4.87E-07
CYP2C9-sub	0.31715232	2.92E-09	0.00031068
CYP2D6-inh	8.46E-21	2.63E-16	9.80E-19
CYP2D6-sub	6.36E-12	1.46E-13	0.00015222
CYP3A4-inh	1.83E-10	0.99248213	2.79E-07
CYP3A4-sub	2.47E-11	7.77E-11	8.26E-06
CYP2B6-inh	1	0.99999774	1
CYP2B6-sub	9.12E-35	0	1.18E-36
CYP2C8-inh	1	1	0.99998438
LM-human	2.71E-18	1.82E-05	5.96E-13
cl-plasma	5.54836241	4.62856393	9.57631911
t0.5	4.7994433	4.18491609	3.98295951
BCF	0.97989401	0.94841933	1.21299006
IGC50	3.45854	3.61805825	3.7189326
LC50DM	4.66048773	4.92224985	5.29368527
LC50FM	4.00157249	4.29194152	4.54925651
hERG	0.15848136	0.06947627	0.11006831
hERG-10um	0.89260089	0.99189794	0.8993665
DILI	0.81608784	0.96783441	0.62725943
Ames	0.25883305	0.74022925	0.7121911
ROA	0.22301269	0.4711962	0.61996752

Property	(+)-gallocatechin-(4α→8)-(+)-catechin	Prodelphinidin A2 3-gallate	Proanthocyanidin A-6
FDAMDD	0.99396402	0.98232508	0.9906894
SkinSen	0.99995136	0.99999905	0.99919492
Carcinogenicity	0.00573062	0.03660628	0.02353354
EC	6.39E-07	2.82E-11	3.67E-08
EI	0.85247654	0.12212759	0.79109412
Respiratory	0.82303679	0.40902668	0.70484596
H-HT	0.84677047	0.21430437	0.94438696
Neurotoxicity-DI	0.00033538	6.30E-07	0.00489562
Ototoxicity	0.99705791	0.99986744	0.9949432
Hematotoxicity	0.00332287	3.13E-05	0.01154567
Nephrotoxicity-DI	0.01718301	5.04E-05	0.30954921
Genotoxicity	0.99989438	0.99995089	0.97283882
RPMI-8226	0.00750245	0.00045883	0.04857411
A549	0.99946553	0.99999571	0.98231077
HEK293	0.92300427	0.95261449	0.94446141
NR-AhR	0.12895066	0.00496622	0.08670279
NR-AR	0.00158032	0.00668278	0.02005143
NR-AR-LBD	0.0215649	0.68279457	0.66912037
NR-Aromatase	0.39709401	0.01238423	0.11835982
NR-ER	0.00681893	0.01147423	0.09854289
NR-ER-LBD	0.21136427	0.00098073	0.01222853
NR-PPAR-gamma	0.00065132	9.63E-07	5.13E-05
SR-ARE	0.05949596	0.00017223	0.00260976
SR-ATAD5	0.00010732	1.91E-05	0.01201595
SR-HSE	0.00080828	6.03E-08	3.32E-05
SR-MMP	0.41187295	0.05014908	0.16401516
SR-p53	6.48E-05	5.28E-06	2.67E-05

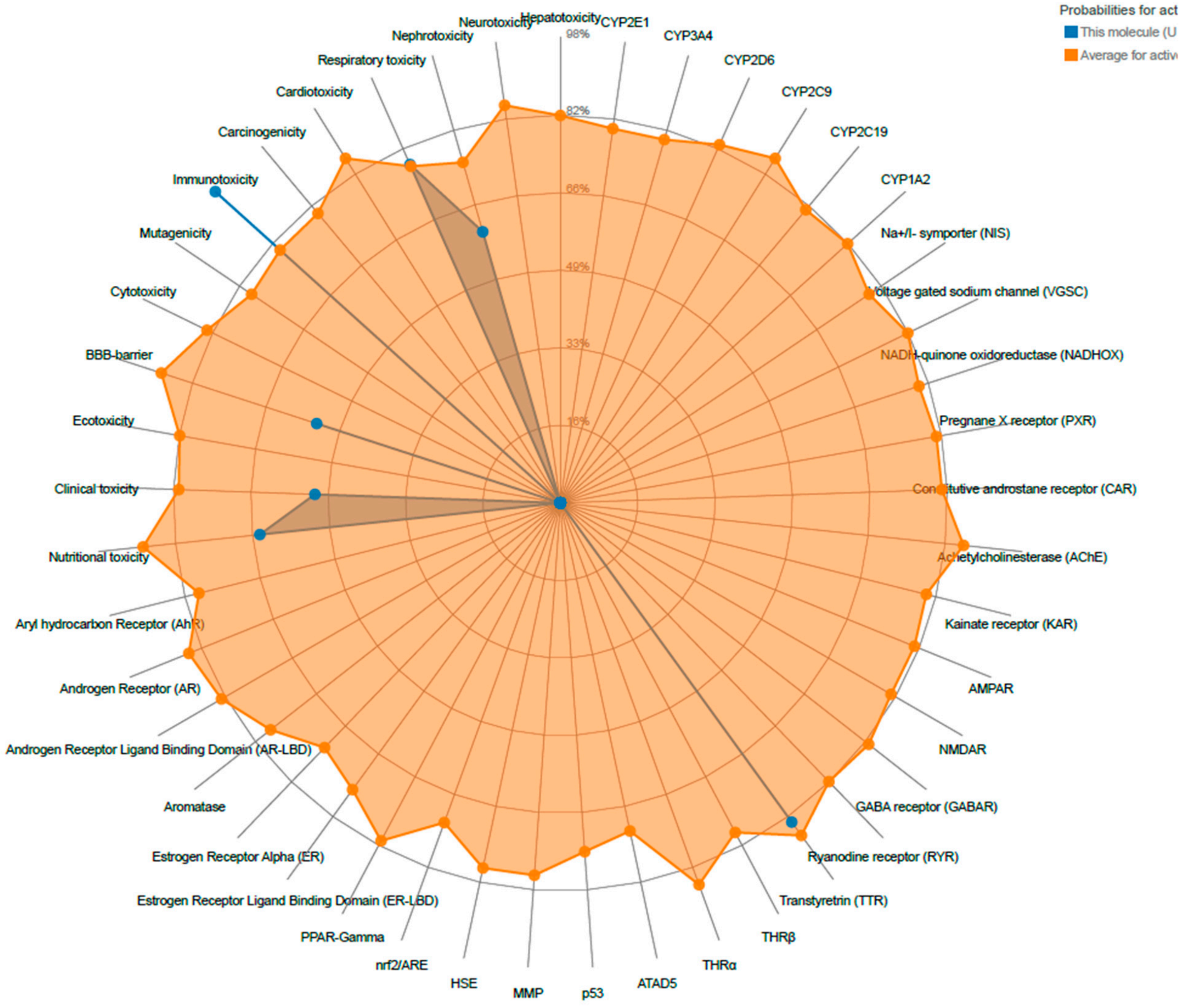

Figure S1: Radar Chart of (+)-gallocatechin-(4α→8)-(+)-catechin.

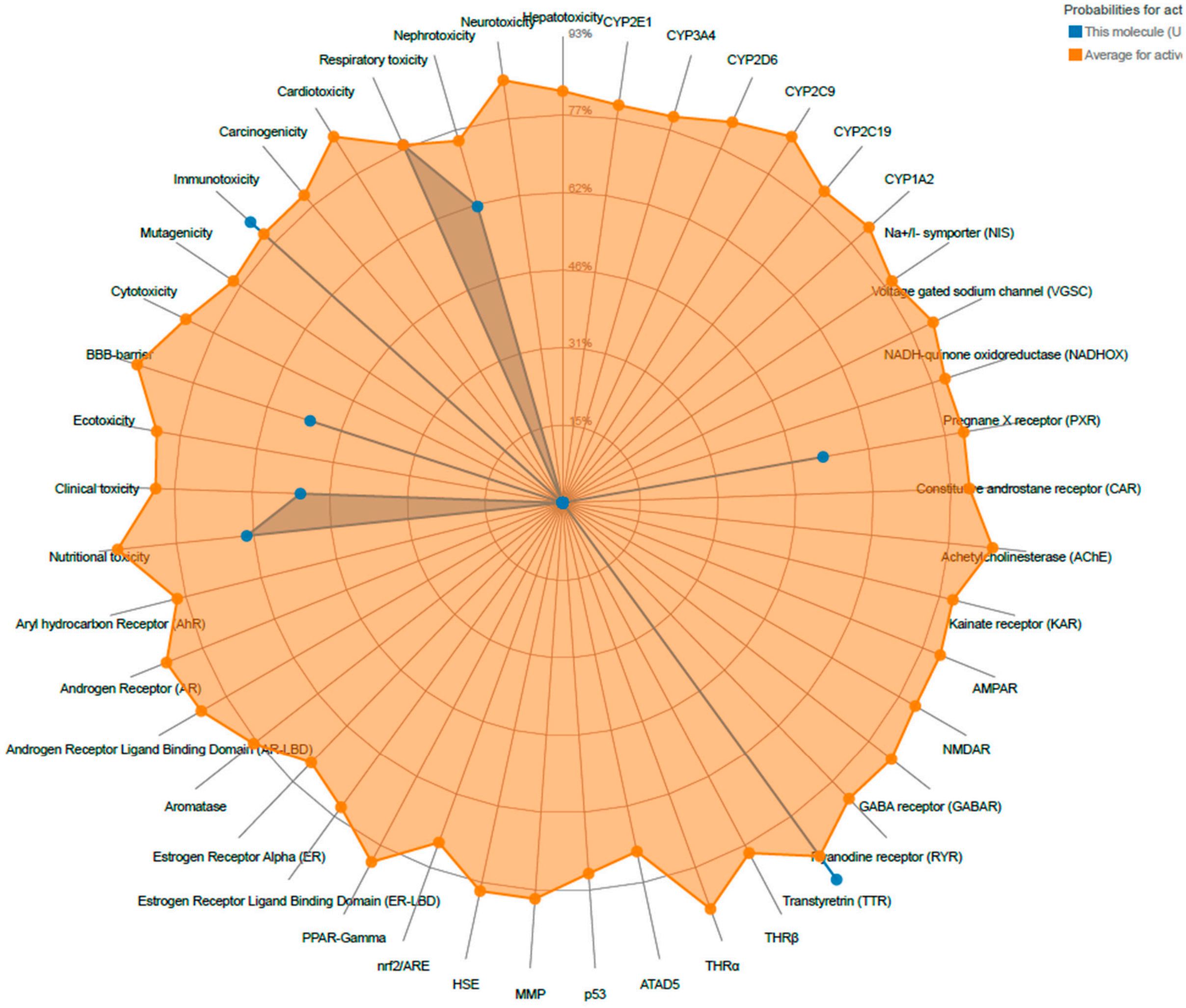

Figure S2: Radar Chart of Prodelphinidin A2 3-gallate.

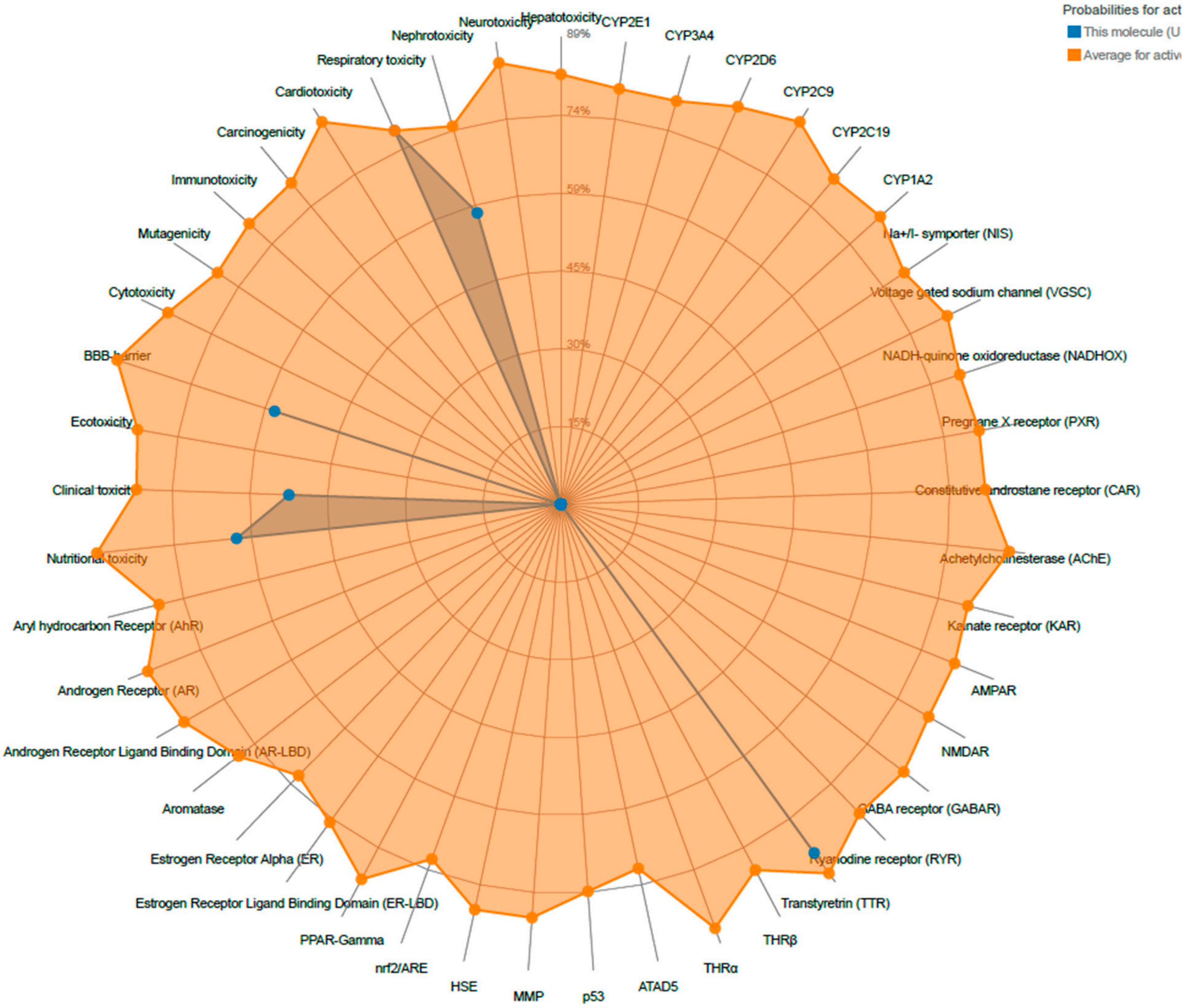

Figure S3: Radar Chart of Proanthocyanidin A-6.

S3. Molecular Docking

S3.1. (+)-gallocatechin-(4α→8)-(+)-catechin

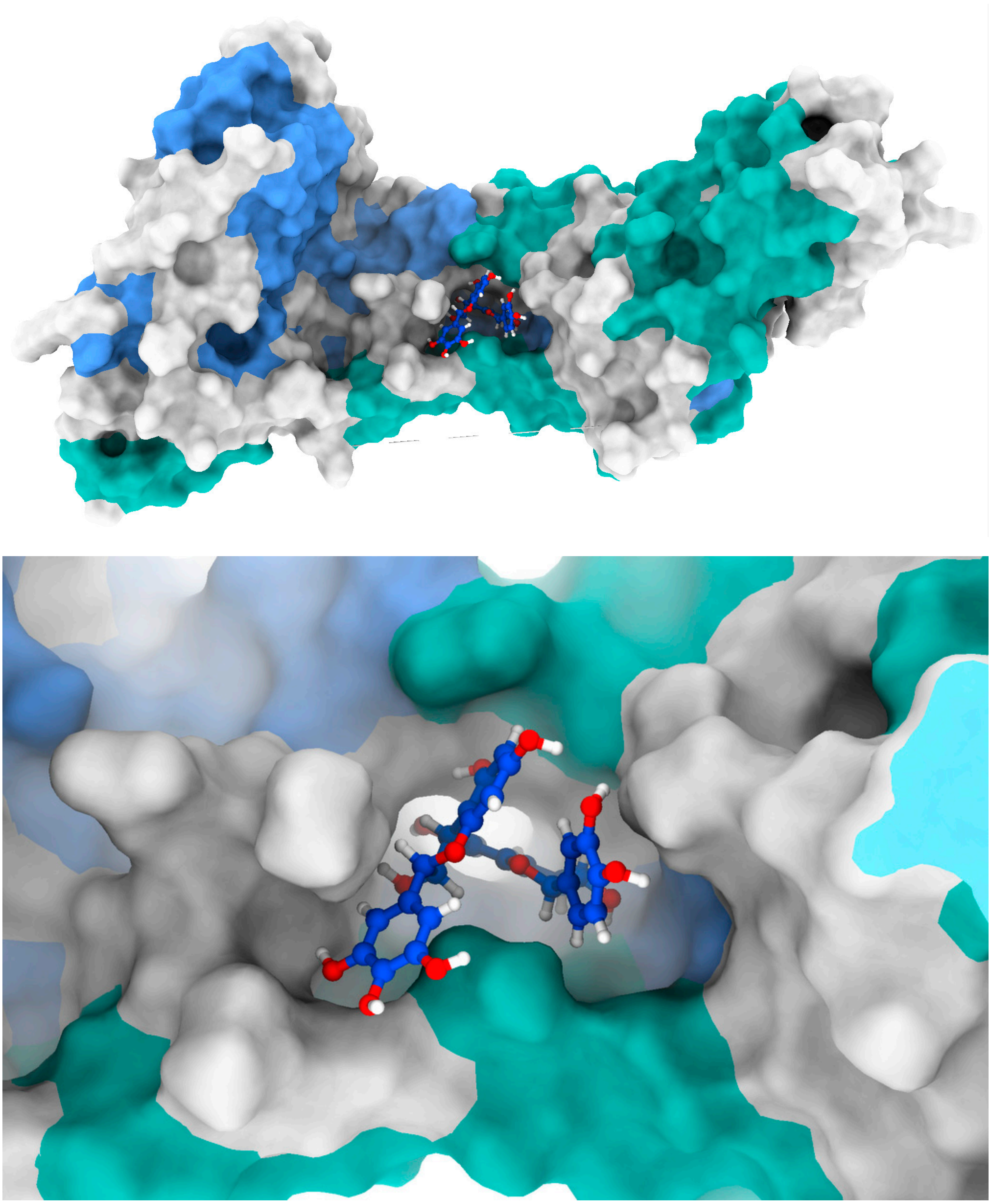

Figure S4: Molecular Docking Visualization of (+)-gallocatechin-(4α→8)-(+)-catechin.

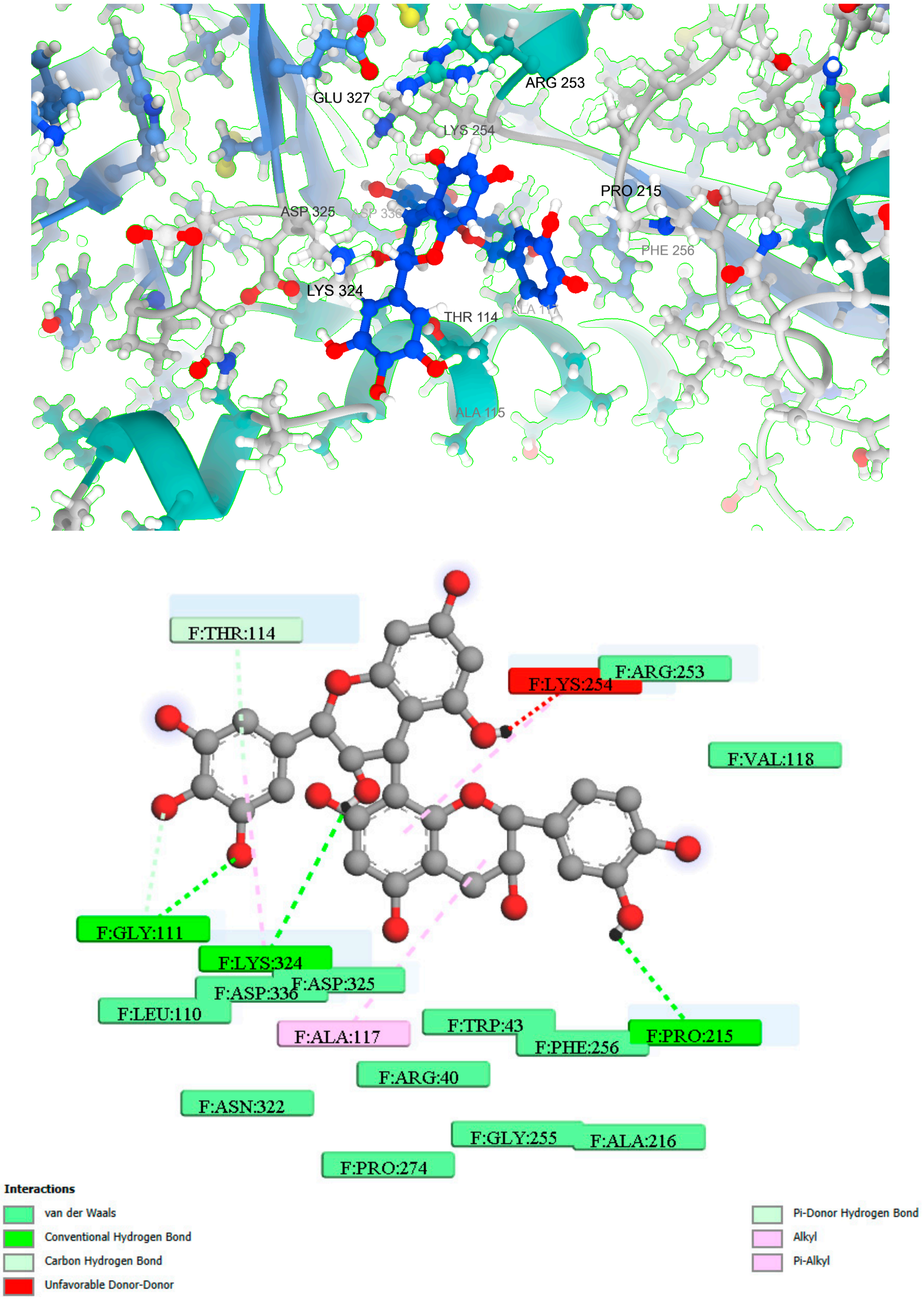

Figure S5: The predicted binding modes and key residue contacts for (+)-gallocatechin-(4α→8)-(+)-catechin.

S3.2. Prodelphinidin A2 3-gallate

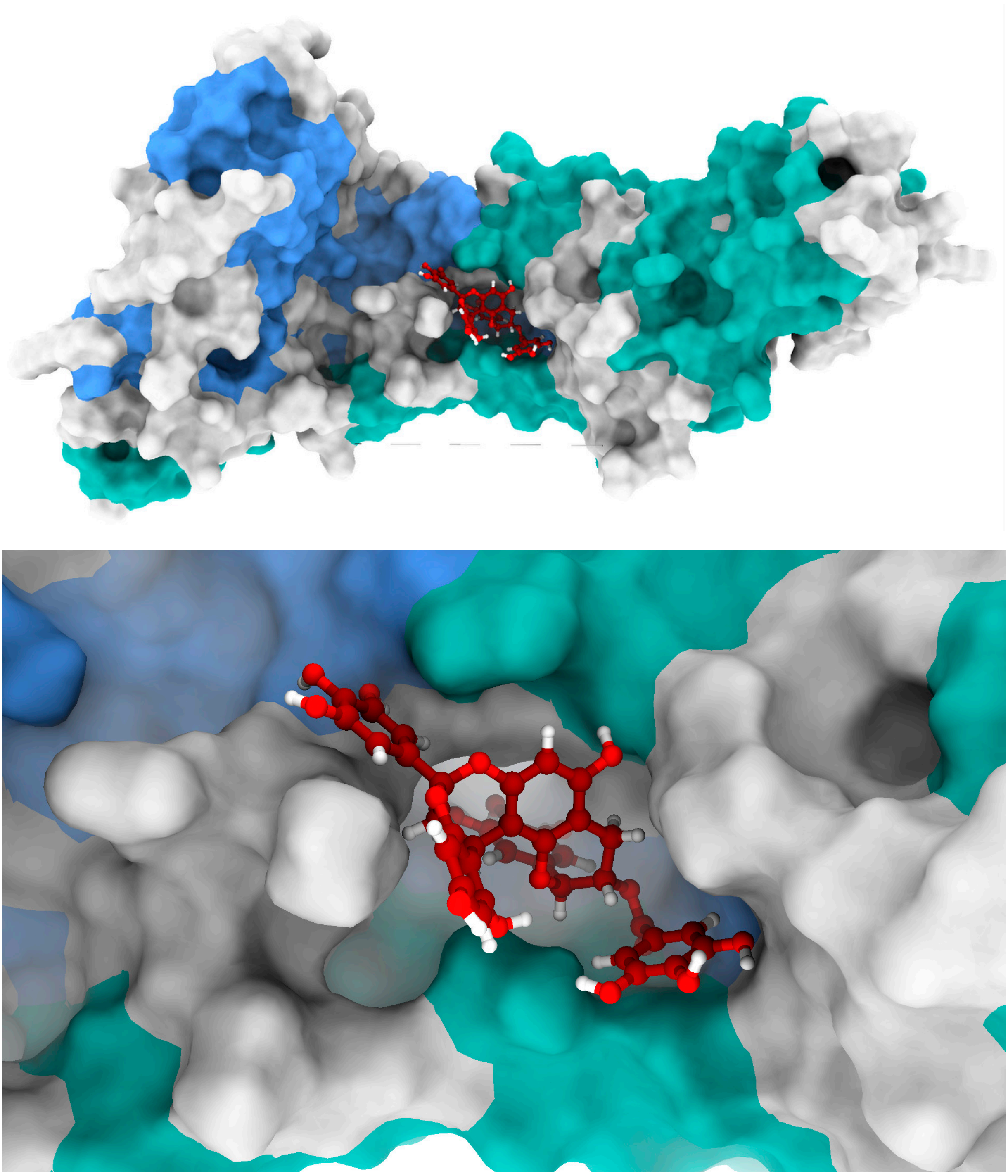

Figure S6: Molecular Docking Visualization of Prodelphinidin A2 3-gallate.

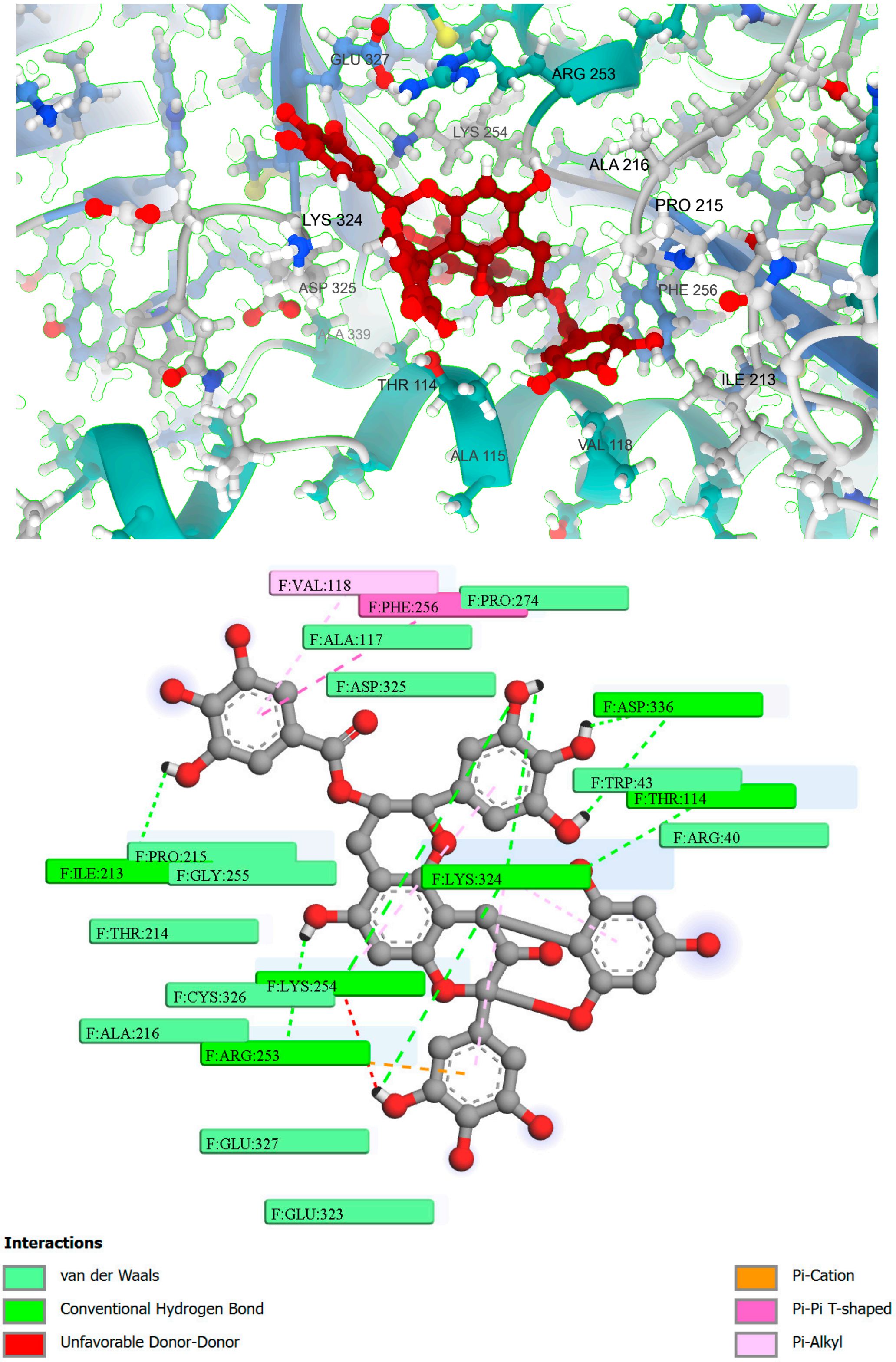

Figure S7: The predicted binding modes and key residue contacts for Prodelphinidin A2 3-gallate.

S3.3. Proanthocyanidin A-6

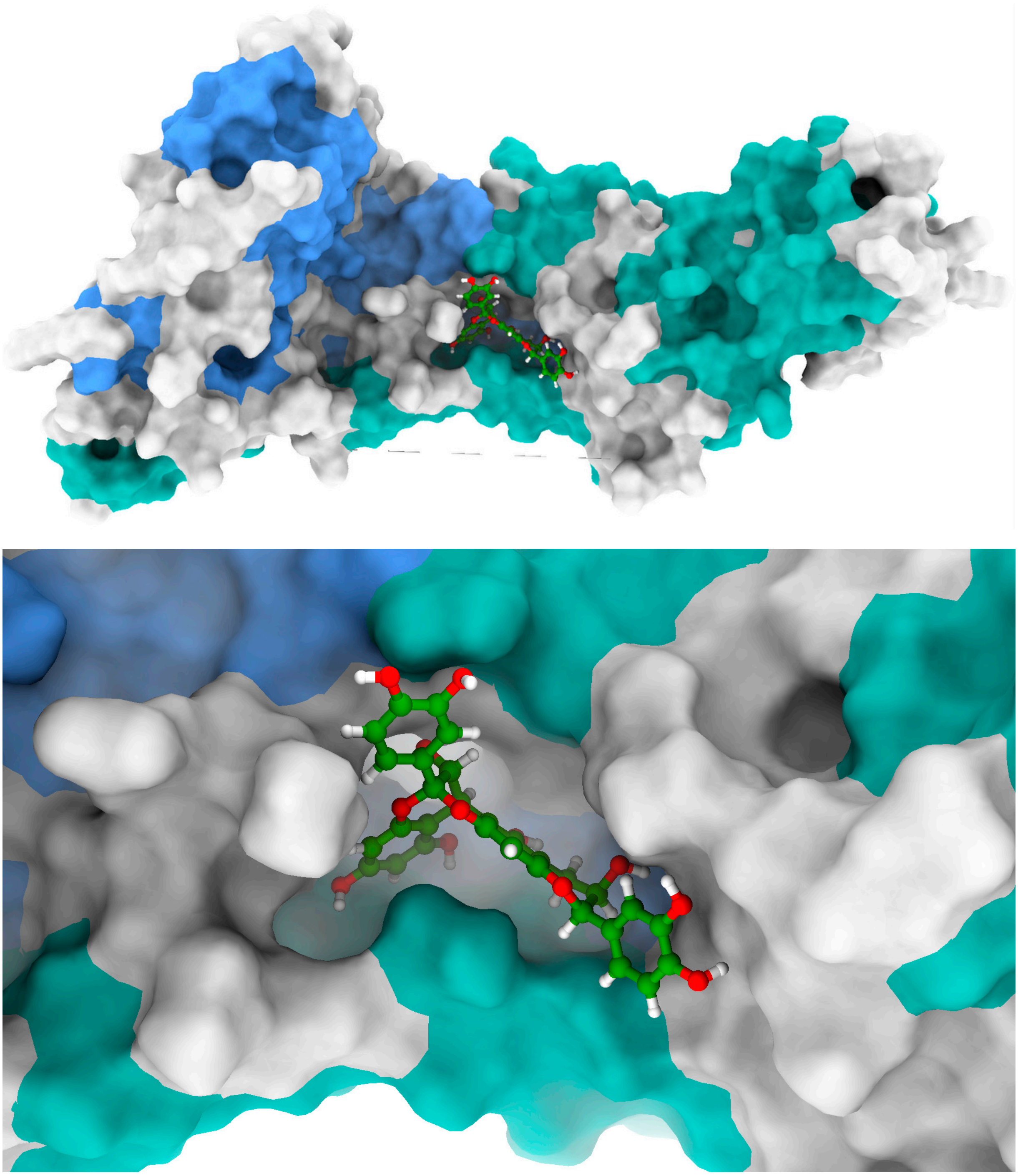

Figure S8: Molecular Docking Visualization of Proanthocyanidin A-6.

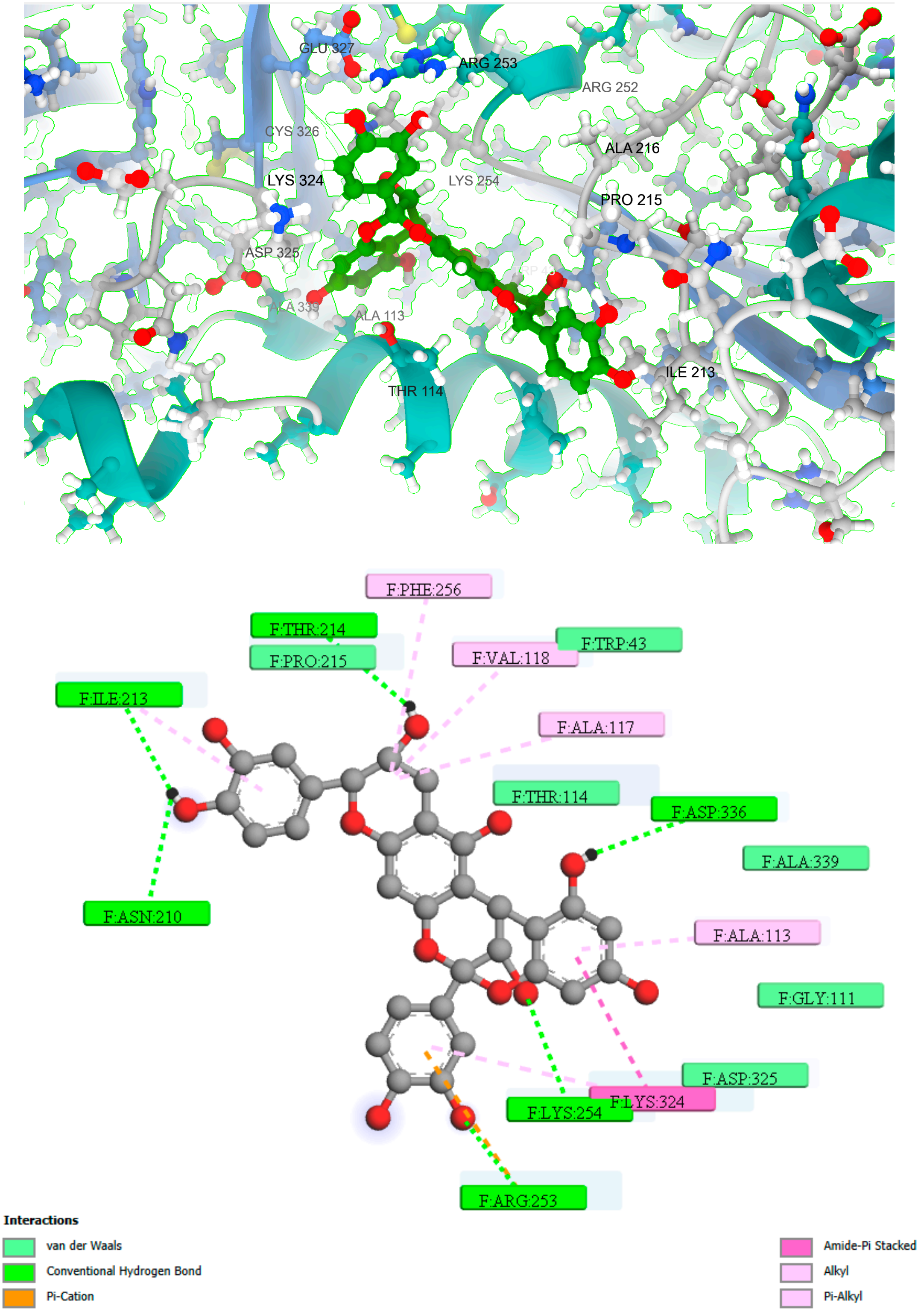

Figure S9: The predicted binding modes and key residue contacts for Proanthocyanidin A-6.

S4. Molecular Dynamics (MD) Simulation

Schrodinger Suite 2024 (v1.6) reports of (+)-gallocatechin-(4α→8)-(+)-catechin, proanthocyanidin A-6, and prodelphinidin A2 3′-gallate
